# Informational stress in cognitive systems: a review and conceptual synthesis

**DOI:** 10.3389/fnetp.2026.1913737

**Published:** 2026-09-21

**Authors:** Elina Asriyan, Ruben Gevorgyan

**Affiliations:** 1 Department of General Psychology, Yerevan State University, Yerevan, Armenia; 2 Chair of Mathematical Methods in Economics, Faculty of Economics and Management, Yerevan State University, Yerevan, Armenia

**Keywords:** allostatic load, attention regulation, cognitive fatigue, cognitive load, information overload, informational stress, rational inattention, network physiology

## Abstract

Modern cognitive environments expose individuals to sustained streams of information that may be complex, uncertain, rapidly changing, or emotionally salient. Such environments can support learning and decision-making but may also require continuing regulation of attention, effort, and information selection. This narrative review examines informational stress as a process-level perspective on the conditions under which sustained informational demands may become stress-related. Drawing on stress appraisal and allostasis, attention and cognitive load, compensatory control, information theory, rational inattention, resource rationality, scarcity, deliberate ignorance, and critical ignoring, we distinguish informational demand from the stress-related response it may generate. Informational stress is defined here as a regulatory process that may arise when sustained informational demands are appraised as taxing and require continued adjustment beyond routine processing. We distinguish this process from information overload, cognitive load, mental fatigue, and specific strategies of selective non-processing. We also outline an operational approach linking structural properties of informational input with appraisal, regulatory responses, behavioral and physiological outcomes, and recovery over time. The perspective is intended as a testable conceptual synthesis rather than a unified theory, and its value depends on demonstrating explanatory contribution beyond established accounts of cognitive demand and regulation.

## 1 Introduction

Cognitive environments of modern days become increasingly characterized by frequent encounters with information that is highly complex, fast-changing, and often emotionally charged. Together, digital technologies, learning software, workplace applications, social media, news outlets, and artificially intelligent content generate circumstances under which people constantly need to choose, filter, interpret, and dismiss information. Whereas access to information has been thought to boost learning, decision-making, and adaptation, research on information overload shows that there is much more to information than meets the eye: information helps perform tasks and understand things, but it can also trigger overload, fatigue, attention fragmentation, disengagement, stress, and poor decisions (Eppler and Mengis, 2004; Speier et al., 1999; Arnold et al., 2023).

Thus arises a central psychological challenge: How do cognitive systems adapt to informational pressures that they face? This question goes beyond the simple matter of whether one could process more or less information. Rather, it deals with how organisms control their limited cognitive, attentional, motivational, and physiological capabilities in response to informational demands imposed upon them. Information may increase attention, effort, and performance in some cases but reduce flexibility, performance, attention, and engagement in others. Understanding this diversity calls for adopting an approach that would consider information both as input and adaptive pressure.

A number of studies have investigated aspects of the problem outlined above. Stress theory, starting with the General Adaptation Syndrome described by Selye, explains how organisms respond to chronic demands through stages of mobilization, adaptation, and exhaustion (Selye, 1950). Later work, in particular the introduction of the concepts of allostasis and allostatic load, highlights the fact that adaptation is dynamic and costly; systems maintain their functions by changing their regulatory status, but the repetition or chronic nature of adaptation may carry significant physiological or psychological costs (McEwen, 1998; McEwen and Wingfield, 2003). These ideas are highly relevant to rich information settings, but classical stress theories were developed without consideration for the structural properties of information or for mechanisms of cognitive filtration of the latter, or for the costliness of processing information.

Another critical literature comes from the domains of cognitive psychology and the learning sciences. Attention and effort models highlight the idea that cognitive capacity is limited, and that task demands have to be in balance with the capacity in order for performance to occur (Kahneman, 1973; Sweller, 1988; Paas et al., 2003). Compensatory control models show that individuals are able to maintain their performance at a high level under high load through increased regulation efforts, although these regulation efforts are costly when they are sustained (Hockey, 1997; 2013). A third set of theories emerges from information processing theory, economics, and behavioral decision-making literature. Rational inattention theory formalizes the idea that information processing is costly and that individuals selectively pay attention to only part of available information (Sims, 2003; Maćkowiak et al., 2023). Scarcity and bandwidth theories suggest that the limitations in cognitive resources lead to selective attention to salient objects at the expense of other objects and goals (Mullainathan and Shafir, 2013; Mani et al., 2013).

The current review brings these strands of research together through the concept of informational stress. We use this term not to characterize information itself as inherently stressful, nor to equate high information load with a stress response. Rather, informational stress refers to an adaptive regulatory state that may arise when sustained informational demands are appraised as taxing available regulatory resources and therefore require continued cognitive, affective, or physiological adjustment (Lazarus and Folkman, 1984; McEwen, 1998). The relevant stressor is thus not “information” in the abstract, but an informational demand whose effects depend jointly on stimulus characteristics—such as complexity, uncertainty, salience, density, or rate of change - and on appraisal, task relevance, controllability, prior knowledge, and available resources. Moderate informational demands may support engagement, learning, and goal-directed effort, whereas persistent demands that require prolonged regulation may contribute to overload, fatigue, attentional narrowing, or disengagement.

In other words, the current literature review will be less ambitious than developing a general theory on cognition under information overload. Specifically, the problem that will be considered is: In what conditions does the presence of persistent information-related requirements give rise to the need for regulatory resources as an adaptive coping strategy under stressful conditions? For the resolution of this problem, the theoretical approaches which can shed light on the four aspects of this process will be used. It is important to note that the intention is not to create a coherent theory on the basis of all these approaches, but rather to show how they allow one to distinguish informational demand from informational stress and to understand in what conditions adaptation to the former becomes a costly experience. The solution of this problem will continue in the following sections of this paper.

The approach taken in this paper uses a narrative and concept-driven review rather than systematic-review approach. The selected literature speaks to different facets of the identified problem: representations of persistent informational needs, evaluation of those needs, regulation of the needs and their translation to stress or cognitive effects. Thus, the review focuses on stress evaluation and allostatic adaptation, cognitive effort and compensatory control, cognitive load, theories of information structure, rational and resource-rational inattention models, and studies of selective or deliberate non-processing of information. The goal is not to cover the literature comprehensively but to contrast their explanations and look for areas of overlap or difference.

## 2 Stress and adaptation under sustained demand

First, the concept of informational stress requires careful incorporation into general stress theory. Information-enriched environments cannot be seen merely as causes of stress as a negative emotional experience or an overload threshold. Stress theory concerns ways that the organism reacts to the demands, maintains functioning, and pays the costs when adaptation needs to be sustained.

From this point of view, Selye’s General Adaptation Syndrome (GAS) is a good historical precedent, but not a model of cognitive adaptation. GAS describes the chronology starting with mobilization (alarm stage), continued by functioning with demand persistence (resistance stage), and finishing with deterioration due to exhaustion of the adaptive processes (exhaustion stage) (Selye, 1950). The usefulness of the presented approach for our purposes is related to the availability of a wide chronology of terms, rather than the suggestion of biological stages of cognitive adaptation to informational load. We do not assume that cognitive response to informational load follows any biological stages or is inevitably associated with exhaustion.

However, Selye’s theory cannot be considered as a complete theory of stress since it was elaborated to explain physiological reactions to stress, rather than to provide insight into the cognitive processes during informational adaptation. Namely, the General Adaptation Syndrome does not incorporate such aspects of cognitive and affective appraisals as threat assessment, management of emotional experiences, motivational aspects, attention allocation, and the structure of information received (Lazarus and Folkman, 1984). These elements should be accounted for in the framework of informational stress. Therefore, GAS has been used only to describe general patterns of mobilization, regulation, and possible collapse; however, the processes involved in stress arising from information must be explained by means of appraisal, regulation, and adaptation.

One of the main advancements of stress research beyond the General Adaptation Syndrome is the concept of allostasis. While homeostasis concerns stability provided by relatively stable internal set points, allostasis refers to the ability of the organism to stabilize its functioning through changing its regulatory mechanisms as a response to external demands (Sterling and Eyer, 1988; McEwen, 1998). From this perspective, the adaptation to an information-enriched environment can be regarded as a series of changes that allow the subject to keep functioning. It is important to recognize the need for allostasis because cognitive systems operate differently from physiological systems and the adaptation process is much more dynamic.

Allostatic load is especially applicable to informational stress, since this concept refers to the overall cost that is related to multiple and prolonged regulation changes. While allostasis is defined as the process of maintaining stability via adaptation, allostatic load is a term that refers to a cost that might occur due to multiple activation of regulations, prolonged duration of regulations or insufficient recovery after regulation (McEwen and Stellar, 1993; McEwen, 1998). Informational load can be rather small if taken into account separately–it could be a message, notification, interruption or need for making a choice. However, informational demands that are recurrent, overlapping, ambiguous or emotional might require persistent regulation efforts from individuals. Individuals have to regulate their attention, inhibition processes, updating expectations and making choices about how to react on information (Boksem and Tops, 2008; Hockey, 2013). In other words, the value of allostatic load concept does not consist in comparing cognitive effort and physiological stress, but in demonstrating the potential cumulative cost of adaptations in case of insufficient recovery.

Allostatic load provides another connection between the physiological and psychological perspectives since even cognitive strains can be reflected in a physiological mode. Subjective experiences like cognitive fatigue and diminished functioning can be accompanied by stress- and allostasis-related brain-body changes (McEwen and Gianaros, 2011), including increased heart rate variability, electrodermal activity, pupil dilation, hormonal changes, and effort-related brain responses (Kim et al., 2018; van der Wel and van Steenbergen, 2018). Although such factors are not a direct measure of informational stress, they can become elements of multimodal assessments of allostatic load related to informational demands.

Thus, one can see how the transition from General Adaptation Syndrome to allostatic load facilitates our comprehension of how stress theory can be adapted to informational stress. While Selye suggests three temporal phases of adaptation to stressors, allostatic theory introduces the idea of stability through changes. Moreover, the concept of allostatic load emphasizes the cost of repeated adjustments needed to adapt to the information-enriched environment.

## 3 Cognitive resources, effort, and load

Stress-adaptation approaches also explain why ongoing demands require adaptation and can entail costs. More specific, and therefore important, theories emphasize attention, working memory, effort, control, and performance. Since cognitive systems do not perceive or process incoming information passively and unlimitedly, such processes require the deployment of finite cognitive resources, which need to be allocated, protected, and sometimes withdrawn.

For example, the attention and effort approach proposes the resource-based view of attention, where attention is seen as an internal capacity or a resource that needs to be allocated between ongoing activities (Kahneman, 1973). While effort means mobilizing attention resources to fulfill demand, if task requirements are low, then available capacity allows performing the activity with minimum subjective strain. However, when task requirements are higher, additional effort ensures performing the activity satisfactorily. In addition, if there is a lack of capacity for addressing the demands (when demands are high or competition for resources between tasks is strong), then it causes performance deterioration. Thus, this perspective illustrates why informational stress cannot be equated to the number of stimuli since the same information demands may have different costs depending on the relevance of the tasks, uncertainty, time pressure, familiarity, motivation, *etc.* The same dense text may be comfortable for the expert but stressful for the beginner, while a brief text may still cause effort expenditure in case it is urgent and ambiguous.

However, recent approaches have focused on explaining cognition as an adaptive management of limited resources rather than as an intrinsic limitation on the level of capacity. According to the theory of resource rationality, the way cognitive mechanisms operate can be interpreted as a tradeoff between the potential advantages of higher precision of information processing and its costs in terms of the limited availability of time, memory, attention, and computing power (Griffiths et al., 2015; Lieder and Griffiths, 2020). Hence, from the perspective of resource rationality, the use of simpler strategies of processing, simplification, and selective information processing do not necessarily mean any failure of cognition but may be an appropriate response to the cost of computing. The approach seems particularly relevant to the concept of informational stress since it moves the discussion away from the mere existence of limited resources to their regulation within the given environment. Yet, while resource rationality explains what adaptive allocation of cognitive resources means, it does not tell us when it triggers a stress response or produces costs.

Another widely known theory–cognitive load theory–is oriented toward designing effective learning experiences in instructional contexts (Sweller, 1988; Paas et al., 2003; Sweller, 2023). It emphasizes that working memory has limited capacity, which should not be exceeded when creating information loads that help learners acquire knowledge or skills. In particular, the theory differentiates between intrinsic, extraneous, and germane load, which illustrates that not all information load is harmful. For example, there needs to be enough difficulty or stimulation in order for the learner to focus on the task, while extraneous demands may cause information overload in cases of poor structuring, high redundancy, fast presentation, *etc.* Thus, this theory highlights how structural, contextual, and task characteristics define whether informational demands are useful or burdensome.

This perspective has limitations in the context of informational stress research. First, it is mainly concerned with instructional design and learning, rather than other domains where informational demands are experienced. Second, it does not emphasize accumulation or gradual regulation of demands, but rather explains immediate information processing. While one can understand from this theory why someone can experience information overload in the course of performing a single learning task, the process of experiencing informational stress through prolonged engagement remains unknown. This limitation brings up the concept of compensatory regulation in information environments.

Compensatory control theory illustrates how people are able to cope with high workloads by means of using various strategies that allow maintaining adequate performance. Thus, in case information processing requires effort, people can compensate for their inability to address demands actively, thus preventing performance deterioration (Hockey, 1997). Specifically, a person may allocate more effort, more resources, or use an alternative strategy in order to maintain performance, and it does not always happen immediately. Breakdown happens not due to initial demand but because of failing to cope with it anymore (Hockey, 2013). For instance, while in information-rich settings, a student may stay concentrated throughout the challenging part of the text, a worker may perform well in terms of receiving multiple messages, and a decision-maker may analyze all necessary information, these behaviors rely upon effort compensation, which leads to fatigue later on.

Together, these theories suggest that the effect of informational demand on performance may well have a non-monotonic profile as opposed to being either strictly positive or negative. Low demand may result in under-engagement, while moderate demand may improve one’s attention and effort, with excessive or continuous demand being associated with overload, fatigue, and even behavior simplification. This broad inverted U-shaped curve can be compared with classic arousal-performance relations exemplified by the Yerkes–Dodson curve and related work on adaptive gain and stress-based decision making (Yerkes and Dodson, 1908; Aston-Jones and Cohen, 2005; Starcke and Brand, 2012). In this context, the inverted U shape is used as a qualitative guideline as opposed to an absolute quantifiable rule since it depends on many factors, including task-specific considerations, personal factors, and the nature of demand. Thus, informational demand is not necessarily harmful as moderate demand can be productive while persistent and poor informational demand may incur some costs.

## 4 Selective information processing: information theory, rational inattention, and scarcity

It takes more perspectives to explain what drives a cognitive system to process particular information selectively and disregard other data. Information theory, rational inattention, and scarcity-based explanations of bandwidth each provide their vantage points on this question. Together, they offer a picture of informational environments as structured realms marked by uncertainty, cost, relevance, and constraint.

Information theory is a formal language in which stimuli are characterized as statistical phenomena. Shannon’s conception of entropy was designed to describe uncertainty in the context of communication systems, and it had nothing to do with meaning, interpretation, or relevance (Shannon, 1948). This distinction should be emphasized. In the present discussion, the use of entropy and its various types, like conditional entropy or spectral entropy, as proxies of semantic and psychological properties will not be assumed. Instead, such concepts will be explored as possible proxies of structural unpredictability and variability in the stimulus.

Such an approach is productive because different informational environments can indeed vary in their statistical characteristics. A display may contain several conflicting stimuli; a text may feature unknown words; an interface may frequently change or produce signals of different kinds. Information-theoretic variables, including entropy, may help in characterizing such environments. Research on perceptual and cognitive processing indicated early interest in the relationship between information theory and human sensory experience, particularly in vision and neural encoding (Attneave, 1954; Barlow, 1961). Further theoretical development extended information-theoretic and probabilistic approaches to language processing and attentional allocation (Hale, 2001; Levy, 2008; Tatler and Vincent, 2009), while related work has used variability and complexity measures to characterize neural signal dynamics (McIntosh et al., 2008).

Thus, informativeness of the stimuli is one factor that contributes to informational load, but it is not the only one. Entropy may capture structural features, like variability or predictability, but not semantics, emotions, prior knowledge, motivational importance, or task relevance. A complex stimulus may not be relevant to the ongoing task and ignored as a result. Conversely, a simple message may cause distress if perceived as threatening. Thus, the relation between stimulus structure and cognitive effort has to be mediated by attention regulation, appraisal, tasks, and cognitive capabilities. In other words, informational load cannot be reduced to the mathematics of entropy alone.

The notion of information being costly is captured by the idea of rational inattention. According to traditional economic theory, an agent tends to process all available information before making a decision. The theory of rational inattention assumes that agents face a limited capacity for attention, and information processing costs resources (Sims, 2003; 2010). Therefore, in addition to choosing actions, agents also choose the amount of information to be acquired and processed. Subsequent research has generalized the concept to include such ideas as bounded rationality and discrete choice under informational costs (Matějka and McKay, 2015; Woodford, 2020; Maćkowiak et al., 2023).

From the above perspective, selective processing is a response to costs of attention. If the environment produces an excess of information, ignoring some part of it can be the optimal choice. Individuals can focus on relevant stimuli, sacrifice accuracy, resort to simplified decision-making procedures, categorize information, and apply various rules of thumb. Informational inattention can thus be viewed as an adaptive strategy rather than a defect.

In more recent psychological studies, this view is extended through the recognition of different types of adaptive non-processing. Deliberate ignorance refers to a deliberate decision not to gain access to information when the revelation of such information is likely to have certain psychological or emotional costs (Hertwig and Engel, 2016). In relation to the digital context, the concept of critical ignoring similarly focuses on the selective disregard of poor quality, deceptive, or irrelevant information as a form of cognitive skill, rather than inattention (Kozyreva et al., 2023). The relevance of such concepts to informational stress lies in the fact that filtration and disengagement do not always come from one specific source but can be driven by different factors.

Rational inattention, deliberate ignorance, and critical ignoring explain the adaptiveness of selective non-processing but are not all of the regulatory strategies covered by this paper. Rational inattention formalizes selective information gathering in terms of processing costs, whereas deliberate ignorance and critical ignoring highlight conscious choices about what not to know or pay attention to. Some other withdrawal strategies may not be strategic in nature but develop incrementally with increasing demand that coincides with fatigue, lack of control, or inability to maintain effort. For that reason, the informational stress perspective considers non-processing a diverse set of regulatory strategies that need to be distinguished both in purpose and in source.

Finally, the theory of scarcity can also provide some insight. As argued by Mullainathan and Shafir, scarcity captures the attention by focusing it on deficits and preventing consideration of other issues (Mullainathan and Shafir, 2013). Experimental studies on the effect of poverty on cognitive functioning show that concerns about resource shortages consume cognitive capacity and impair cognitive performance (Mani et al., 2013). Later reviews discussed resource scarcity as an analytical tool to explain how people make decisions and allocate attention under scarcity (de Bruijn and Antonides, 2022). Although this literature focuses not on informational complexity but resource scarcity, it may still be useful due to the way it affects cognitive processes.

The concept of cognitive tunneling may be especially useful. Scarcity causes individuals to concentrate intently on urgent problems, ignoring less immediate but equally important priorities. While such behavior is adaptive in the short term, it can become costly and limit flexibility in decision-making and information integration. A similar pattern may arise in the context of informational stress, whereby constant, urgent, or emotionally salient information streams lead to tunneling attention towards immediate threats, alerts, or rewards while ignoring the rest of available information.

Information theory, rational inattention, and resource scarcity theory converge on the same point: information cannot be processed unconditionally. Filtering, compression, prioritization, and disengagement are necessary steps. These operations are usually effective and adaptive, but they become problematic under prolonged stress due to rigidity, excessive simplification, tunneling, and avoiding the source of stress altogether.

## 5 Informational stress as an integrative perspective

The theoretical traditions reviewed above address different but complementary aspects of adaptation to demanding informational environments. Stress and allostatic approaches emphasize appraisal, regulation, and cumulative cost; attention, cognitive-load, and compensatory-control accounts explain effort and performance under limited capacity; information-theoretic approaches characterize selected structural properties of informational input; resource-rational and rational-inattention approaches describe adaptive allocation of limited processing resources; and scarcity, deliberate ignorance, and critical ignoring highlight forms of selective attention and non-processing. The informational stress perspective does not treat these traditions as components of a single theory. Its narrower purpose is to organize their contributions around one process-level question: when do sustained informational demands require regulatory adjustment of a kind that may reasonably be characterized as stress-related and potentially costly?

Informational stress is defined here as a regulatory process that may arise when informational demands are sustained, appraised as taxing (Lazarus and Folkman, 1984; McEwen, 1998; Hockey, 1997; 2013), and require continued adjustment of attention, effort, or information-processing strategy. This definition distinguishes informational stress from informational demand itself. An informational demand is a property of the interaction between an individual and an information-rich environment and may reflect complexity, uncertainty, density, rate of change, ambiguity, urgency, or emotional salience. Such a demand does not constitute stress merely because it is information-rich. Its effects depend on appraisal, task relevance, controllability, prior knowledge, motivation, and available regulatory resources. Informational stress therefore begins not with the presence of information, but when coping with the demand requires sustained regulation beyond routine processing.

There are four elements to distinguish: informational demand, appraisal, regulatory response, and outcome. The reactions to the demand may consist in paying attention and effort, filtering, prioritizing, simplifying, ignoring, tunneling, avoiding, and disengagement (Sims, 2003; Maćkowiak et al., 2023; Mullainathan and Shafir, 2013; Mani et al., 2013). The reactions are not necessarily maladaptive and can allow one to cope with the demand if it is not very high. The outcomes can be successful coping, narrowing of the attention scope, fatigue, inflexibility, overload, or withdrawal if there is a persistent attempt to cope with the demand or insufficient recovery. Therefore, informational stress is not associated with any particular behavior or physiological indicator. Informational stress is expected to be recognized from the complex picture of the demand, its evaluation, adjustment to it, and its outcomes.

Consistent with the narrow scope assigned to the GAS in Section 2, it might be useful to refer to the concepts of alarm, resistance, and exhaustion to identify general time-based patterns in response to sustained information load. The initial load can trigger arousal of attention and efforts, continuous load requires some form of regulation through filtering, prioritization, and simplification, and failure to sustain regulation can lead to poor performance, avoidance, or disengagement in the end. It must be noted that these sequences of responses do not have to be viewed as biologically determined stages. The latter pattern makes more sense in terms of accumulated regulatory costs and lack of sufficient recovery, consistent with allostatic load theory (McEwen, 1998; Hockey, 2013).

In this way, informational stress perspective does not replace existing theories but combines them. The value of the approach is in identifying a common point: adaptive regulation is necessary when demands are extended in time, and the results of the adaptation may be positive and negative. Table 1 highlights the place of all literature in the theory synthesis.

**TABLE 1:** Contributions of reviewed theoretical traditions to the informational stress perspective.

Theoretical tradition	Main contribution	What remains underspecified	Contribution to informational stress
General adaptation syndrome	Stress as temporal mobilization, resistance, and exhaustion	Cognitive appraisal, meaning, and information structure	Provides a heuristic temporal template for adaptation under sustained demand
Allostasis and allostatic load	Regulation through change with cumulative cost	Specific mechanisms of attention and filtering	Explains why sustained regulation may become costly
Attention and effort	Limited resources allocated under demand	Long-term adaptation and stimulus structure	Explains effort mobilization and cost of sustained processing
Cognitive load theory	Working-memory limits and instructional design	Stress dynamics beyond learning contexts	Explains how complexity and poor structure produce overload
Compensatory control	Performance maintenance through increased effort	Formal stimulus representation	Explains delayed breakdown under sustained demand
Information theory	Formal description of uncertainty and structure	Meaning, appraisal, and stress response	Provides possible measures of stimulus informativeness
Rational inattention	Selective processing under information costs	Fatigue, affect, and cumulative burden	Explains why ignoring information may be adaptive
Deliberate ignorance and critical ignoring	Intentional decisions not to acquire or attend to particular information	Non-deliberate fatigue-related withdrawal and cumulative stress-related consequences	Explains adaptive forms of intentional non-processing and information filtering
Scarcity and bandwidth	Tunneling under constrained resources	Stimulus complexity and informational structure	Explains attentional narrowing under constraint

### 5.1 Boundary conditions and distinctions

Nevertheless, informational stress can be conditional. Not all difficult information triggers it. And not any stress impacts performance negatively. The first factor is the task relevance: If it is clear that certain complex information has anything in common with achieving an aim, it may help to focus and learn better. However, when such information is irrelevant, people may experience cognitive distraction and extra cognitive load (Sweller, 1988; Mayer, 2021). The second condition relates to the duration of exposure to information: A small time interval allows activating effort. However, long exposure to it is more likely to result in fatigue or avoidant behavior (Hockey, 2013). At the same time, individual conditions also matter: knowledge, resilience, motivation, attention control, and emotional state may influence how information is perceived.

Moreover, information organization matters too. Well-structured information helps in reducing uncertainty and making cognitive processing easier. But when the information is ambiguous or too fast-changing, or, on the contrary, too redundant and slow-changing, additional regulatory efforts are needed. Communication of risks, conflict, or assessment of one’s worth or status is emotionally more engaging, and hence imposes higher regulatory loads than purely informative information. In other words, the impact of information depends not only on its complexity but also on the level of emotional involvement in the communication.

Information overload, cognitive load, mental fatigue, and informational stress represent different stages in the process of adaptation. Information overload represents a situation when the amount, complexity, or speed of information exceeds an individual’s capacity for effective processing or leads to a deterioration of task performance (Eppler and Mengis, 2004; Arnold et al., 2023; Dote Pardo, 2026). Cognitive load is a concept that relates to the strain on a person’s working memory while engaging in activities (Sweller, 1988; Paas et al., 2003). Mental fatigue is a condition that may develop after an extensive cognitive load and be reflected in subjective fatigue and changes in motivation or performance (Boksem and Tops, 2008; Hockey, 2013). What distinguishes informational stress from the aforementioned notions is that it has a process-oriented perspective and involves appraisal, regulation, and possible behavioral, subjective, or physiological consequences of prolonged informational demands. Therefore, one might experience informational stress in cases when there is no information overload or performance impairment due to the temporary maintenance of performance through regulatory processes.

Informational stress needs to be differentiated from models explaining a certain regulatory process. In rational inattention and resource-rational approaches, there is an attempt to model the allocation of limited informational or cognitive capacities in a selective manner with the cost of processing in mind (Sims, 2003; Maćkowiak et al., 2023; Griffiths et al., 2015; Lieder and Griffiths, 2020), while deliberate ignorance and critical ignoring involve conscious decision-making related to not acquiring certain information (Hertwig and Engel, 2016; Kozyreva et al., 2023). While all these processes can take place under informational challenges, their existence does not mean the presence of informational stress. On the contrary, informational stress does not presuppose the optimality and intentionality of selective processing, and regulation can include compensation efforts, involuntary attentional narrowing, avoidance, and disengagement as the demands continue. Hence, the suggested construct involves the process connecting informational demands, appraisals, regulation, and outcomes.

The conceptual structure described above is summarized in Figure 1.

**FIGURE 1:**
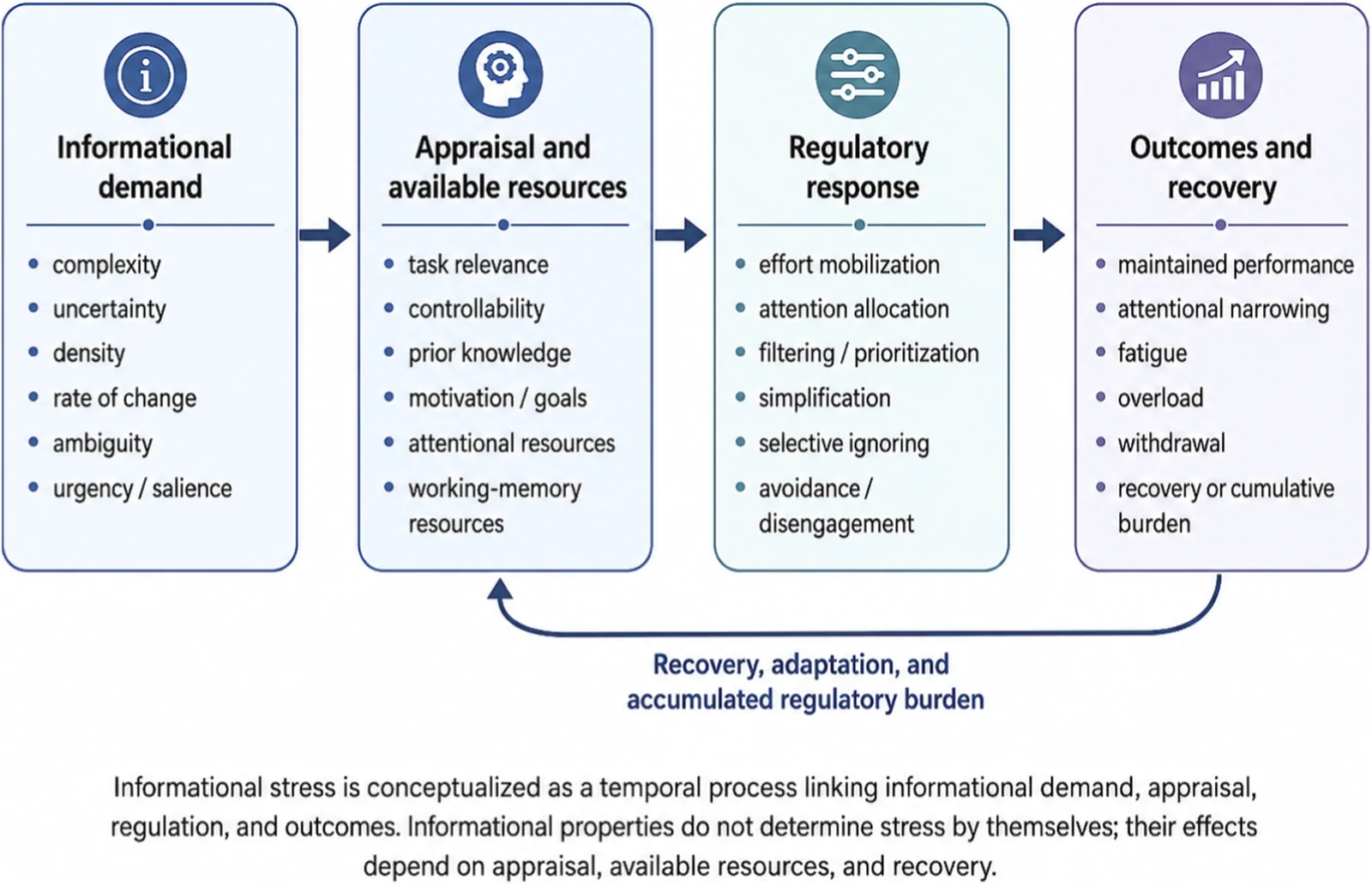
Conceptual model of informational stress. Informational stress is defined as a process linking four constructs: informational demand, appraisal and resources, regulatory processes, and outcomes. Informational demands may differ in their degree of complexity, uncertainty, density, changeability, ambiguity, urgency, and salience, but all of them do not define stress by themselves. It depends on whether the demands are relevant to the task, controllable, what information the person knows before, how motivated he/she is and how many cognitive resources he/she possesses. The regulation includes effort expenditure, attention allocation, filtration, prioritizing, simplification, ignoring, avoiding or disengaging. The outcomes vary from continued performance and adaptation to narrowed attention, fatigue, overload, or withdrawal. Recovery can help in restoring the regulatory function, while repeated or prolonged regulation without proper recovery can lead to the accumulation of the load. Hence, the model emphasizes the temporal relations between demand, appraisal, regulation, and outcome, not some characteristics of informational stress.

## 6 Future research directions

However, despite its conceptual nature, the informational stress perspective offers clear implications for empirical study. It relies upon researchers’ ability to measure informational demands and associated regulatory responses in different circumstances, in addition to specifying the circumstances in which adaptation continues to be feasible or becomes costly.

First, the informational demands have to be defined through the use of suitable metrics with respect to the nature of the stimulus distribution. The Shannon entropy reflects the average amount of uncertainty in the case of discrete stimulus distributions, whereas the conditional entropy shows the remaining uncertainty when relevant context information is included (Shannon, 1948; Attneave, 1954; Barlow, 1961). In the case of information sequences, the metric entropy rate represents a better description of average uncertainty than the vague term “temporal entropy.” Spectral entropy has to be used for time-dependent stimuli with frequency spectrum, like auditory or physiological stimuli, but not in order to describe the complexity of static texts or display. Concerning the single stimuli, the surprisal indicates the surprisingness of a particular word, signal or event and has been used for measuring the processing costs in psycholinguistic studies (Hale, 2001; Levy, 2008). For visual stimuli, the metrics concerning gaze allocation and scanpaths may be used as additional metrics, demonstrating how the participants select the available information (Holmqvist et al., 2011; Tatler and Vincent, 2009). It has to be noted that the structural metrics have to be combined with the subjective evaluations regarding the complexity, urgency, relevance, controllability and salience of the stimulus.

### 6.1 A minimal operational scheme

A basic operationalization can begin with the identification of the relevant information states of a particular task. Let X be a discrete stimulus variable whose states are xᵢ, and let pᵢ be the probability of each state, estimated, for example, by the relative frequency of occurrence of the state in a certain time window. Shannon entropy, H(X) = −Σ pᵢ log_2_ pᵢ, then represents average uncertainty over those defined stimulus states. For a sequential stream X_1_, X_2_, … , the entropy rate characterizes average uncertainty per observation while taking temporal dependence into account. At the event level, surprisal, −log_2_ p (xᵢ), quantifies how unexpected a particular stimulus is relative to the estimated distribution. For example, in the context of digital monitoring, events can be classified into task updates, alerts, messages, and other stimuli. The frequency of occurrence of each category during subsequent time periods will constitute the distribution used for the calculation of entropy, and each individual stimulus’s surprisal level will represent the level of surprise in a particular case. The definition of relevant states and the observation period should be made in advance because the values of both measures depend on the representation of the informational environment.

Information-theoretic and time-series methods can also be used to characterize statistical dependence and temporal coupling between informational inputs and regulatory responses. Mutual information, I (X; Y) quantifies statistical dependence between a stimulus variable X and a response variable Y; it is symmetric and does not by itself provide information about directionality or causation. For dynamic processes, transfer entropy provides a directed information-theoretic measure by quantifying whether the past of one process contains information about the future of another beyond the information contained in the latter’s own past (Schreiber, 2000). Granger causality constitutes a distinct, model-based predictive framework: in its standard linear formulation, one variable is said to Granger-cause another when its past values improve prediction of the latter beyond what can be predicted from the latter’s own past (Granger, 1969). For multivariate settings, partial information decomposition can distinguish unique, redundant, and synergistic information contributed by multiple source variables about a target variable (Williams and Beer, 2010). In a monitoring task, such methods could be used to examine statistical dependence or temporal coupling between properties of the incoming event stream and subsequent pupil dynamics, heart-rate variability, or behavioral variability. None of these measures establishes informational stress by itself; rather, they provide formal tools for testing statistical dependence and temporal relationships between properties of informational input and regulatory responses.

A concrete experimental design can illustrate how these factors can be incorporated. Take, for instance, a prolonged digital monitoring task wherein individuals watch a flow of information consisting of task-related alerts, warnings, messages, and distractions. While keeping the total volume of events approximately constant, it is possible to manipulate the level of the unpredictability, interruptions, controllability, and task relevance of the information stream. The informational demand will be assessed based on the previously mentioned structural factors together with experimental manipulation. Appraisal will be measured by the perception of urgency, controllability, relevance, and informational demand. The regulation will be measured by changes in the attentional allocation, pupil dilation, reaction time, filtering, and effort perception. The outcomes will include accuracy, fatigue, avoidance, and recovery after the task. In this particular case, informational stress will not necessarily be defined by high entropy, elevated levels of cognitive load, and physiological arousal. The proof of informational stress will come in the form of a converging phenomenon where there is high informational demand that is evaluated as stressful and results in continuous regulation and its outcomes.

Second, the response of regulatory systems to experimental perturbations needs to be assessed using convergent rather than single-measure evidence. Eye-tracking parameters such as fixation durations, gaze allocation, and scanpaths could reveal shifts in sampling of information-relevant and information-irrelevant stimuli (Holmqvist et al., 2011). Pupillary dilation might signal mental effort and cognitive control demands (Beatty, 1982; van der Wel and van Steenbergen, 2018), while heart rate variability might add information about autonomic control under stress-related conditions (Kim et al., 2018). Indices obtained through electroencephalographic (EEG) measures such as shifts in alpha band activity and fronto-theta power would provide additional indices of attention and cognitive-control demand (Klimesch, 1999; Gevins and Smith, 2003). The indices of behavioral measures like response time variability, error rates, filtering behavior, task-switching, avoidance, and decreased persistence would further refine the picture of the shift in regulatory processes. None of these measures is specific for informational stress; they can signal cognitive load, effort, fatigue, arousal, and others processes as well. Recent findings from mental fatigue studies also demonstrate heterogeneous neurophysiological responses, making the multimodal assessment more promising compared to relying on a single biomarker (Goodman et al., 2025). In the context of the proposed approach, these measures become informative when assessed in conjunction with the independently identified informational stressors and their temporal dynamics.

Given the temporal dynamics of the phenomenon of interest, longitudinal designs appear to be especially fitting. One could use experimental manipulation of stimulus complexity, informational rate, ambiguity, emotional valence, or task relevance, along with collection of behavioral and/or physiological data. Comparison might include the contrast between structured vs. unstructured materials with otherwise similar information, variation in interruption rates in digital tasks, and experimental modification of informational uncertainty in decision-making tasks. Attention and vigilance paradigms would also be relevant, given that the maintenance of sustained attention and vigilance involves effort and may eventually result in cognitive decline (Warm et al., 2008; Hopstaken et al., 2015).

A more refined prediction would pertain to the contribution of structural characteristics of the informational stream toward regulatory demand apart from the volume of the stimulus and the nominal difficulty of the task. Apart from accounting for the number and frequency of presented events and the nominal difficulty of the task, any difference in terms of the statistical and temporal structure of the informational stream—such as the predictability and temporal dependence of the stream—should affect regulatory demand. In addition, the impact of these structural characteristics of the stream should be moderated by appraisal processes, such as the perceived relevance and controllability of the task. Hence, even in cases where two tasks involve the presentation of the same number of events and require the same level of performance, there could be differences in the regulatory demands because one stream could be more unpredictable, while the contents of the task are considered to be relevant and difficult to control. Information-theoretic approaches provide formal ways to assess statistical unpredictability and temporal dependence, while stress-appraisal models consider relevance and controllability.

However, the theoretical framework may still be subject to disconfirmation. The value of the theory will be greatly undermined if, despite accounting for task complexity, amount of information and measures of cognitive load and effort (Kahneman, 1973; Sweller, 1988; Paas et al., 2003), differences in information structure and evaluation are unable to predict regulatory response and recovery. Similarly, the validity of the theory will be weakened if the posited model of informational stress from demand, through evaluation and regulation to result cannot be empirically separated from the existing theories on cognitive load, compensatory control, and mental fatigue (Hockey, 1997; 2013; Boksem and Tops, 2008). The aforementioned considerations have practical importance since informational stress seeks to describe a unique process by which the properties of the information environment give rise to prolonged adaptation and regulation, and not just to provide a novel term for cognitive tasks.

Overall, the present framework can be applied to many research domains. In education, it can contribute to understanding how learning materials might be designed in order to maximize richness without causing excessive cognitive load (Sweller, 1988; Mayer, 2021). In HCI, it may help develop interfaces that balance informativeness against excessive information density and reduce interruptions. In organizational psychology, it may illuminate how digital communication tools help and hinder workers; recent evidence links information overload and fear of missing out in the digital workplace with stress, burnout, and poorer mental health (Marsh et al., 2024). With respect to AI-based technologies, it may inform the design of generative systems capable of summarizing and organizing information, while at the same time increasing informational load.

## 7 Scope, limitations, and conclusion

The informational stress can be seen as conceptual integration across multiple scientific traditions without aiming at producing a unified theory of cognition, stress response, or decision-making. Informational stress should not be confused with information processing in general; the former implies a need for sustained cognitive regulation, whereas the latter can be routine and pleasant. Moreover, moderate information demands can engage cognitive processing and facilitate performance. In particular, informational stress relates to information-theoretic measures of structure rather than its content or subjective significance.

First, it is important to emphasize the limits of application of Shannon’s entropic metric. Entropy and related measures describe structural uncertainty but not meaning, relevance, subjectively experienced value, or emotions. Second, despite the similarity of terminology used by biologists and information scientists, biological stress models cannot serve as an exact template for information-related adaptive responses because of substantial differences between psychological, cognitive, and physiological stress responses (Selye, 1950; McEwen, 1998). Finally, while rational inattention describes selective processing of information in economics, it should not be considered equivalent to regulation of informational stress in psychology. Economists consider rational inattention as a formal theory of efficient information processing (Sims, 2003; Maćkowiak et al., 2023).

This review is narrative and conceptually guided rather than systematic. Its purpose is to compare theoretical traditions relevant to sustained informational demand and adaptive regulation, not to provide exhaustive coverage of all empirical studies in the areas of stress, information overload, cognitive load, rational inattention, scarcity, or mental fatigue. Consequently, the selection and interpretation of the literature necessarily reflect the conceptual scope of the review, and other relevant traditions or empirical findings may not be represented. The framework should therefore be regarded as a theoretically motivated synthesis whose value depends on whether its proposed distinctions and predictions prove useful in subsequent empirical work.

As is evident nowadays, information-rich environments are ubiquitous to modern learning, working, social interactions, communication, decisions, and other processes associated with cognitive adaptation and performance. It is important not merely that individuals face much more information today, but they also have to constantly regulate their attention towards information and disengage when necessary.

Accordingly, the contribution of the informational stress perspective to the existing knowledge is the synthesis of findings related to the following question: How do individuals adapt to information demands? Information itself is not necessarily stressful for cognitive systems; instead, informational environments can become challenging when they require cognitive effort and regulation due to certain qualities of information. Thus, while information can mobilize the person’s efforts and improve his or her cognitive performance in the immediate period, persistent information demands eventually lead to adaptation.

Overcoming informational demands requires filtering, ignoring some information, simplifying complex stimuli, engaging in selective attention (tunneling), or even disengagement from informational sources. These adaptive responses might be successful in initial phases, but they can later become maladaptive when they reduce cognitive flexibility, prevent further learning, or cause fatigue and information avoidance.

In order to develop an informational stress perspective further, future studies should be conducted dynamically and multi-modally by considering factors such as information stimuli, their evaluation and adaptation by the individual, attentional behavior, physiological responses, and performance indicators.
